# Case of Lead Poisoning Associated with Herbal Health Supplements

**DOI:** 10.5696/2156-9614-10.28.201214

**Published:** 2020-12-07

**Authors:** Abbas Ali Mahdi, Jamal Akhtar Ansari, Avinash Agarwal, M. Kaleem Ahmad, Suhail Sarwar Siddiqui, Tabrez Jafar, Thuppil Venkatesh

**Affiliations:** 1 Department of Biochemistry, King George's Medical University, Lucknow, India; 2 Department of Chemistry, Shibli National PG College, Azamgarh, India; 3 Department of Critical Care Medicine, King George's Medical University, Lucknow, India; 4 Department of Personalized and Molecular Medicine, Era University, Lucknow, India; 5 St. John's Medical College, Bengaluru, India

**Keywords:** lead poisoning, blood lead levels, herbal health supplements

## Abstract

**Background.:**

Lead poisoning is a chronic health condition arising from prolonged ingestion and exposure to lead above permissible limits. Although reported globally, developing countries like India and neighboring countries are amongst the most affected by lead.

**Objectives.:**

The aim of the present study was to evaluate lead poisoning associated with herbal health supplements in a suspected case.

**Materials and Methods.:**

A 31-year-old male reported consuming sixteen different herbal health supplements. The case and supplements were assessed for lead levels. The patient came from one of the metro cities of Uttar Pradesh state, India.

**Results.:**

The blood lead level of the case was found to be 78.40 μg/dL, which was much higher than the permissible limit of ≤5 μg/dL. Moreover, one of the supplements was found to have a very high lead content.

**Conclusions.:**

The present study demonstrated a case of lead poisoning which was very likely due to high lead content present in one of the supplements. The case had typical neurological signs of lead toxicity such as irritability, frequent headache, mental dullness, generalized pain, muscle weakness, numbness and tingling, and twitching and shaking of the legs while sleeping.

**Patient Consent.:**

Obtained

**Competing Interests.:**

The authors declare no competing financial interests.

## Context

Lead poisoning is a form of toxic metal poisoning in humans caused by overexposure to lead. Lead interferes with a variety of body processes and is toxic to many organs and tissues including the heart, bones, intestines, kidneys, and reproductive and nervous systems.^1^ It interferes with the development of the nervous system and is therefore particularly toxic to children, causing potentially permanent learning and behavioral disorders. Symptoms include abdominal pain, confusion, headache, anemia, irritability, and in severe cases, seizures, coma, and death.^2–5^ With deleterious effects on all organs in the human body, lead poisoning is widely recognized as a major public health problem globally. Children and women are particularly vulnerable to lead poisoning, especially in developing countries.^6,7^ Many health hazards are preventable with appropriate laws and regulations, and lead poisoning is mostly preventable when appropriate regulations are in place.

Worldwide, the major sources of lead are lead-based batteries, lead-based paints, gasoline additives, food cans, chain soldering, ceramic glazes, drinking water systems and cosmetic and folk remedies.^8,9^ Human exposure to lead occurs through various sources like leaded gasoline, industrial processes such as lead smelting and coal combustion, lead-based paints, lead-containing pipes or lead-based solder in water supply systems, battery recycling, grids and bearings, *etc*.^10,11^ However, one of the most unexplored routes of lead poisoning is herbal-based medicines. The Centers for Disease Control and Prevention reported that from 2000 to 2003, there were 12 cases of lead poisoning in adults associated with Ayurvedic medication intake in five different locations within the United States.^12^ Moreover, some of the Ayurvedic preparations and supplements which are commonly used for body building have been found to contain lead and/or mercury at 100 to 10,000 times over acceptable limits.^13^ Without sufficient public awareness, the risk of heavy metal exposure in individuals taking these supplements is quite high and concerning. The present study presents a case of lead poisoning associated with herbal health supplements.

## Case Presentation

In October 2019, a 31-year-old male amateur bodybuilder approached the authors with the complaint of fatigue for unknown reasons, numbness of the palms and soles, irritability, frequent headache, mental dullness, generalized pain, muscle weakness and tingling, as well as twitching and shaking of the legs while sleeping. A detailed patient history revealed that the patient had been taking herbal supplements for the last three years. The patient consulted a general practitioner who referred him for testing at a private pathology laboratory in New Delhi. Test results revealed that his blood lead level (BLL) was 59.43 μg/dL. Thereafter, he contacted the National Referral Centre for Lead Projects in India (NRCLPI), King George's Medical University, Lucknow, India. The examination at NRCLPI showed that his BLL was 78.4 μg/dL. In addition, it was also observed that the patient had an elevated serum creatinine level (between 1.8 to 2.1 mg/dL) and he reported having foamy urine over the previous one month. The complete analysis results are shown in Supplemental Material.

### Analyses

Blood analyses revealed that the hemogram, liver and renal function tests of the patient were normal except for a few parameters. Metal analysis was carried out by inductively coupled plasma-optical emission spectrometry (ICP-OES) after microwave digestion of samples as described by Ansari *et al.*, 2015 with a slight modification.^14^ Briefly, whole blood and different types of herbal supplements were acid digested in a microwave reaction system with a temperature and pressure sensor (Multiwave 3000, Anton Paar, Perkin Elmer, USA). The resultant clear solution was analyzed by ICP-OES using a low flow aqueous system (Perkin Elmer, Optima, 8000, USA).^14^ On metal analysis his BLL was found to be 78.40 μg/dL (Normal: <5 μg/dL). We also investigated the lead content of different herbal supplements which the patient was taking for over three years and the results are summarized in Table 1.

**Table 1:** Lead Levels in Different Herbal Health Supplements

**Herbal health supplements**	Results* (ppm)
Supplement #1	1.59
Supplement #2	1.89
Supplement #3	ND
Supplement #4	0.94
Supplement #5	ND
Supplement #6	ND
Supplement #7	0.80
Supplement #8	ND
Supplement #9	ND
Supplement #10	ND
**Supplement #11**	**9265.97**
Supplement #12	0.84
Supplement #13	ND
Supplement #14	ND
Supplement #15	ND
Supplement #16	ND

^*^World Health Organization limit for herbal formulations, 10 ppm 18

**Abbreviations**
*BLL*: Blood lead level
*NRCLPI*: National Referral Centre for Lead Projects in India

### Differential diagnosis- herbal supplements induced lead toxicity

After the patient reported to NRCLPI and his BLL was found to be 78.4 μg/dL, he was directed to immediately stop the consumption of all herbal supplements.

### Outcome and follow-up

Overall, the patient's BLL trended down over time after proper nutritional uptake and stopping of herbal supplementation. After stopping the consumption of herbal preparations, beginning a diet rich in fruits and vegetables, and beginning adequate supplementation of iron, zinc and calcium, the patient's BLL came down to 15 μg/dL. This level does not require any chelation therapy. Chelation at first instance was not advised due to known adverse effects of chelation therapy.^15^ His symptoms resolved after six months of diet modification and essential mineral supplementation, and his last BLL was 15 ug/dl. The heavy metals investigation of these supplements showed that one of the formulations had a very high lead concentration (9265.97 ppm) *(Table 1).* The patient reported using 16 different types of formulations for approximately three years as health supplements.

## Discussion

The present study presents a case of chronic lead poisoning associated with herbal supplements. Currently, hundreds of different herbal formulations are available, online as well as over-the-counter, for body building and to treat a wide range of illnesses including the common cold, diabetes, infertility, cardiovascular problems, diabetes, etc.^13,16–17^ Despite the therapeutical properties and natural origin of herbal formulations, several foreign materials and toxic metals such as lead, mercury, arsenic, etc. can accumulate in these herbs from environmental sources and other anthropogenic activities such as mining, dry and wet deposition of industrial effluents and agricultural activities and domestic sewage.^18^ A comprehensive analysis of 193 Ayurvedic medications revealed the presence of heavy metals in approximately 20% of products analyzed.^13^ In the present case, the patient reported using 16 different types of formulations over a period of three years as a health supplement. The heavy metal investigation of these supplements showed that one of the formulations had a very high lead concentration (9265.97 ppm). The lead level was found to be 900 times higher than the limit of lead content as specified by the World Health Organization (WHO).^19^ The supplement was primarily used for muscle building and increasing testosterone level. The very high content of lead present in the supplement very likely led to the patient's lead toxicity.

The present case had complaints of fatigue, headache, mental dullness, muscle weakness, numbness and tingling, and twitching and shaking of legs while sleeping of unknown etiology. The lead poisoning in the present case was established after estimation of blood lead levels. Blood lead level is the most convenient and readily available biomarker for probable lead poisoning/toxicity. However, BLL has a limitation to a certain extent as it may not necessarily depict the total body burden or exposure to lead. Prolonged exposure of lead results in accumulation in bones, teeth and many other tissues such as the brain, spleen, kidneys and liver. There have been reports showing that lead creates reactive oxygen radicals at the cellular level which may lead to alterations in DNA and cell structures; moreover, lead also interferes with enzymes of vitamin D synthesis. In addition, lead mobilization from bone may interfere with intracellular calcium signaling and has a detrimental effect which may lead to increased bone turnover.^20,21^

In the present case, we observed that the BLL of the patient was initially 78.4 μg/dl and after one month it was reduced to 59.43 μg/dl, and further reduced to 54.3 μg/dl after three months. Moreover, we observed that after stopping the source of lead and beginning supplementation with iron, zinc and calcium along with increased consumption of a nutritious diet, this led to a further significant reduction of BLLs. There have been reports that adequate supplementation of iron and calcium may decrease lead absorption, and zinc restores the activity of some enzymes and vitamin C may increase renal excretion.^22^ In the present case we avoided chelation therapy as chelators act as metal antagonists and form coordination compounds which prevent or reverse the binding of essential metallic cations to body ligands.^15^

Blood investigations showed that the patient had elevated serum creatinine, which may be associated with a possible disturbance in renal function. There are reports that high BLLs can impair renal function, as estimated mainly through serum creatinine levels and rates of creatinine clearance from the body.^23,24^

Overall, the present discussion raises the alarm of lead poisoning associated with herbal health supplements which are commonly used in body building. Moreover, the Indian Society for Lead Awareness and Research (InSLAR) has been working in this area for the last several years and has issued a white paper on this problem. (Unpublished data).

## Conclusions

Lead poisoning is a life-threatening condition associated with severe acute and chronic complications. The case presented here raises serious concerns that unregulated domestic and foreign herbal health medications/supplements are a potential public health hazard. The present case also demonstrated that more stringent regulations are needed from government agencies along with further scientific research and validation for heavy metal contamination in herbal supplements, to ensure public safety. In addition, community-based awareness programs about lead poisoning are needed.
