# Supplementary material for: Case of Lead Poisoning Associated with Herbal Health Supplements
Source: J Health Pollut. 2020 Dec 7;10(28):201214. doi: 10.5696/2156-9614-10.28.201214 (PMC7731492; doi:10.5696/2156-9614-10.28.201214)
Supplement: Supplementary file 1 [file Mahdi_Supplemental.doc]

**Supplemental Material**

| **Functions/Tests** | **Parameters** | **Results** | **Reference range** |
| --- | --- | --- | --- |
| **Kidney function test** | Uric acid serum | 5.80 | 3.50-7.20 mg/dL |
|  | Serum creatinine | **1.22** | 0.67-1.17 mg/dL |
|  | Estimated glomerular filtration rate (Egfr) | 83 | mL/min/1.73 m^2^  >=90 : Normal |
| **Liver function test** | Alkaline phosphatase | 96.1 | 45 – 129 U/L |
|  | Bilirubin- Direct | 0.19 | < 0.3 mg/dL |
|  | Bilirubin- Total | 0.62 | 0.3-1.2 mg/dL |
|  | Bilirubin (Indirect) | 0.43 | 0-0.9 mg/dL |
|  | Gamma glutamyl transferase (Ggt) | 18.6 | < 55 U/L |
|  | Aspartate aminotransferase (Sgot) | 21.4 | < 35 U/L |
|  | Alanine transaminase (Sgpt) | 26.2 | < 45 U/L |
|  | Protein | 6.7 | 5.7-8.2 gm/dL |
|  | Albumin | 4.1 | 3.2-4.8 gm/dL |
|  | Serum globulin | 2.6 | 2.5-3.4 gm/dL |
|  | Serum albumin/globulin ratio | 1.58 | 0.9 – 2 Ratio |
| **Blood count** | Total leucocyte count | 6.13 X 10³ | 4.0-10.0 X 10³/μL |
|  | Neutrophils | 54.5 | 40-80% |
|  | Lymphocyte | 39.5 | 20-40% |
|  | Monocytes | 3.6 | 0-10% |
|  | Eosinophils | 2 | 0.0-6.0% |
|  | Basophils | 0.2 | <2% |
|  | Immature granulocyte percentage (Ig%) | 0.2 | 0-0.5% |
|  | Neutrophils - Absolute count | 3.34 X 10³ | 2.0-7.0 X 10³/μL |
|  | Lymphocytes - Absolute count | 2.42 X 10³ | 1.0-3.0 X 10³/μL |
| **Vitamins/Miscellaneous** | Homocysteine | 14.6 | <30 µmol/L |
|  | Cystatin C | 0.86 | <1.50 mg/L |
|  | Lipoprotein (A) | **50.3** | <30.0 mg/dL |
|  | 25-OH vitamin D | 43.71 | 30-100 ng/mL |
|  | Vitamin B 12 | 318 | 211-911 pg/mL |
|  | Apolipoprotein-A1 | 124 | 86-152 mg/dL |
|  | Apolipoprotein-B | 92 | 56-145 mg/dL |
|  | APO B/APO A1 RATIO (APO B/A1) | 0.7 | 0.40 - 1.26 ratio |
|  | High sensitivity C-reactive protein (HS-CRP) | 0.2 | <=3.0 mg/L |
| **Hormones** | Amylase | **140.9** | 28-100 U/L |
|  | Lipase | 33.5 | 5.6-51.3 U/L |
| **Electrolytes** | Calcium | 9.34 | 8.8-10.6 mg/dL |
|  | Bun /serum creatinine ratio | 9.57 | 9:1-23:1 Ratio |
|  | Sodium | 137.5 | 136 – 145 mmol/L |
| **Trace elements** | Serum copper | 79.3 | 63.5-150 µg/dL |
|  | Serum zinc | 129.46 | 52-286 μg/dL |
|  | Testosterone | 452.86 | 164.94 - 753.38 ng/dL |
|  | Iron | 104.2 | 65 – 175 μg/dL |
|  | Total iron binding capacity (TIBC) | 385 | 225 - 535 μg/dL |
|  | % Transferrin saturation | 27.06 | 13 – 45% |
|  | Chloride | 101 | 98-107 mmol/L |
| **Heavy metals/Trace elements profiling** | Arsenic | 0.05 | < 0.5 μg/dL |
|  | Cadmium | 0.03 | < 0.15 μg/dL |
|  | Mercury | 0.01 | < 0.5 μg/dl |
|  | Lead | **42.52** | < 15.0 μg/dL |
|  | Chromium | 0.06 | < 3.0 μg/dL |
|  | Barium | 0.16 | < 3.0 μg/dL |
|  | Cobalt | 0.04 | 0.01–0.15 μg/dL |
|  | Cesium | 0.29 | < 0.5 μg/dL |
|  | Thallium | 0.003 | < 0.1 μg/dL |
|  | Uranium | 0.006 | < 0.1 μg/dL |
|  | Strontium | 1.58 | 0.8 – 3.8 μg/dL |
|  | Antimony | 0.42 | 0.01–1.8 μg/dL |
|  | Tin | 0.03 | <0.2 μg/dL |
|  | Molybdenum | 0.06 | 0.07–0.4 μg/dL |
|  | Silver | 0.10 | < 0.4 μg/dL |
|  | Vanadium | 0.04 | < 0.08 μg/dL |
|  | Beryllium | 0.03 | 0.01- 0.08 μg/dL |
|  | Bismuth | 0.01 | 0.01 - 0.08 μg/dL |
|  | Selenium | 18.96 | 6.0 - 34.0 μg/dL |
|  | Aluminium | 0.23 | < 3.0 μg/dL |
|  | Nickel | 0.61 | < 1.5 μg/dL |
|  | Manganese | 1.50 | 0.71 - 2.0 μg/dL |

**Table 2.** Lead Levels in Different Herbal Health Supplements

| **Test/Element** | **Herbal health supplements** | **Results**  **(ppm)** | **Limit (WHO**^18^ **Canada for herbal formulations) (2007) (ppm)** |
| --- | --- | --- | --- |
| **Lead level** | S-1 | 1.59 | **10.0** |
|  | S-2 | 1.89 |  |
|  | S-3 | ND |  |
|  | S-4 | 0.94 |  |
|  | S-5 | ND |  |
|  | S-6 | ND |  |
|  | S-7 | 0.80 |  |
|  | S-8 | ND |  |
|  | S-9 | ND |  |
|  | S-10 | ND |  |
|  | **S-11** | **9265.97** |  |
|  | S-12 | 0.847116 |  |
|  | S-13 | ND |  |
|  | S-14 | ND |  |
|  | S-15 | ND |  |
|  | S-16 | ND |  |
